# Recent progress in the applications of silica-based nanoparticles

**DOI:** 10.1039/d2ra01587k

**Published:** 2022-05-06

**Authors:** A. A. Nayl, A. I. Abd-Elhamid, Ashraf A. Aly, Stefan Bräse

**Affiliations:** Department of Chemistry, College of Science, Jouf University Sakaka Aljouf 72341 Saudi Arabia aanayel@ju.edu.sa aanayl@yahoo.com; Composites and Nanostructured Materials Research Department, Advanced Technology and New Materials Research Institute, City of Scientific Research and Technological Applications (SRTA-City) New Borg Al-Arab Alexandria 21934 Egypt; Chemistry Department, Faculty of Science, Organic Division, Minia University 61519-El-Minia Egypt; Institute of Organic Chemistry (IOC), Karlsruhe Institute of Technology (KIT) Fritz-Haber-Weg 6 76133 Karlsruhe Germany; Institute of Biological and Chemical Systems – Functional Molecular Systems (IBCS-FMS), Director Hermann-von-Helmholtz-Platz 1 Eggenstein-Leopoldshafen D-76344 Germany

## Abstract

Functionalized silica nanoparticles (SiO_2_ NPs) have attracted great attention due to their promising distinctive, versatile, and privileged physiochemical characteristics. These enhanced properties make this type of functionalized nanoparticles particularly appropriate for different applications. A lack of reviews that summarizes the fabrications of such nanomaterials and their different applications in the same work has been observed in the literature. Therefore, in this work, we will discuss the recent signs of progress in the fabrication of functionalized silica nanoparticles and their attractive applications that have been extensively highlighted (advanced catalysis, drug-delivery, biomedical applications, environmental remediation applications, and wastewater treatment). These applications have been selected for demonstrating the role of the surface modification step on the various properties of the silica surface. In addition, the current challenges in the applications of functionalized silica nanoparticles and corresponding strategies to discuss these issues and future perspectives for additional improvement have been addressed.

## Abstract 2

Functionalized silica nanoparticles (SiO_2_ NPs) have attracted great attention due to their promising distinctive, versatile, and privileged physiochemical characteristics.

## Graphical abstract

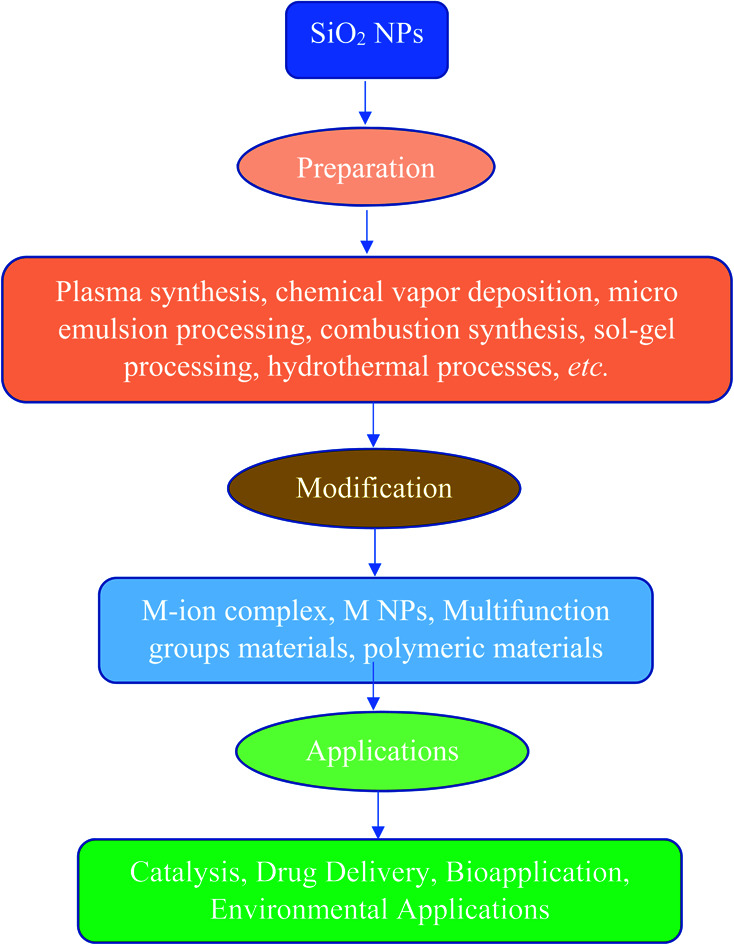

## 1 Introduction

Silica is one of the most abundant components of Earth's crust, and it is naturally produced from various sources such as sugarcane, groundnut shell, corn cobs, wheat straw, rice husk and straw, barley, quartz, olivine, and bamboo stems and leaves. Therefore, many researches have been directed to reuse, reduce, and minimize the hazardous impacts of such agricultural wastes on the environment. Some are widely employed as raw materials in many fields, such as adsorbents in environmental techniques, additives to cement in structural materials, low-cost reinforced filler, and filter materials.^1–3^ Recently, due to the unique properties of silica and its compounds, many works demonstrating the various applications of silica and silica-based materials in different strategic fields have been published.^1^

During the last few decades, nanomaterial technologies have attracted great attention due to their various applications in different fields. Nowadays, due to the developments of unique characteristics of nanoscale particles, nanotechnologies play a vital role in all sciences, engineering, and medical branches. Nanosized silica particles have acquired considerable importance recently.^4^ The promising properties of nanomaterials have obtained considerable attention for various applications such as optoelectronic sensing, agricultural fields, food industries, drug delivery, therapeutic/health, and catalytic technologies. In contrast, nanooxides have other important applications in different fields, including electronics, medicine, cosmetics, food, filler applications, and consumer products.^4–10^ Silica nanoparticles are also considered important nanomaterials having significant applications, which have increased drastically during the last years, have attracted extensive attention, and are widely used due to their greater advantages over conventional nanoparticles; their projected market reached about $8.8 billion in 2020.^9–12^ Many techniques are used to prepare silica nanoparticles (SiO_2_ NPs), such as plasma manufacturing, chemical vapor deposition, microemulsion synthesis, combustion processing, sol–gel synthesis, and hydrothermal processes,^13^ where such fabricated silica nanoparticles are classified into mesoporous and nanoporous nanoparticles. The sol–gel method is considered one of the most important and widely applied techniques utilized to prepare nanoparticles. This method has many advantages, such as synthesis that may be carried out at lower temperatures and desired pH to produce high purity, and the kinetics of their reactions may be controlled by changing the compositions of the reaction mixtures.^3^ During the last decades, mesoporous materials, such as MSNs, have gained great attention from researchers, where it has been considered as one of the most remarkable materials to participate in advanced nanocatalysts, nanosorbents, nanomedicine, and other nanocompounds.^14^

According to the IUPAC system, mesoporous silica nanoparticles (MSNs) structures are classified as particles with pore sizes of 2–50 nm and functionalized with different supplementary groups.^15^ MSNs have had special attention during the last years due to their unique advantages. They have promising characteristics such as beneficial morphological properties, uniform and tunable pore size, porosity, highly chemical stabilities, and attractive physicochemical features that make such nanoparticles strongly attractive in various advanced applications.^8,14,15^ Also, these particles have developed into attractive classes of novel biomaterials due to the biocompatibility of MSNs and their high surface area, pore volume, and considerable surface reactivity.^16,17^

Recently, many works have been investigated to synthesize silica nanoparticles and its nanoarchitectured structure, surface biofunctionality as important porous materials,^3,17^ and directed to control the nanoparticle surface size, morphology, and activity.

**Fig. 1:**
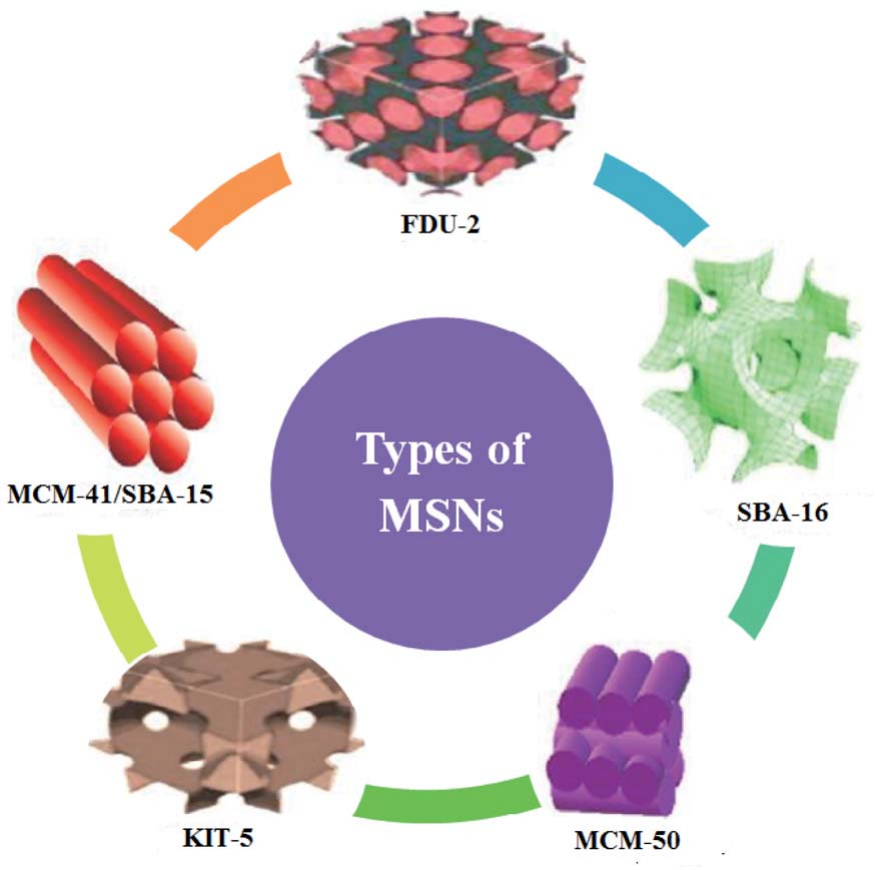
Representation of some of the various types of MSNs (this figure has been adapted/reproduced from ref. 8 with permission from MBDI, copyright 2018).

There are different MSNs such as the M41S family (MCM-48, MCM-41, and MCM-50), SBA-11, SBA-12, SBA-15, SBA-16, KIT-5, COK-12, and FSM-16 with different pore sizes and pore volumes. Many types of these nanoparticles have been developed and utilized in various applications.^14^Fig. 1 illustrates some types of MSNs.^8^

Both MCM-41 and SBA-15 display 2D hexagonal structures and are used for various purposes. Also, some of the MSNs with 3D hexagonal structure (such as SBA-2 and IBN-9) and cubic structure (such as KIT-6, FDU-5, and SBA-16) symmetries have been developed.^17^

Therefore, MSNs have been modified and advanced by different morphologies and structures to act as nanoreactors in industrial catalytic technologies, remediation techniques, and nanocarriers for drug delivery in many biomedical application purposes.^15^

However, the works that reviews and summarizes such nanoparticles' fabrications and their different applications are limited in the literature. Although there have been great reviews discussing certain applications of silica-based nanomaterials separately. Therefore, herein, we will discuss the progress, design, and advantages of silica-based nanomaterials and the applications of these materials during recent years. Major challenges and limitations, current gaps, and the lack of industrialization of most silica-based nanomaterial applications will also be investigated.

## 2 Silica-based nanomaterials and its applications

**Fig. 2:**
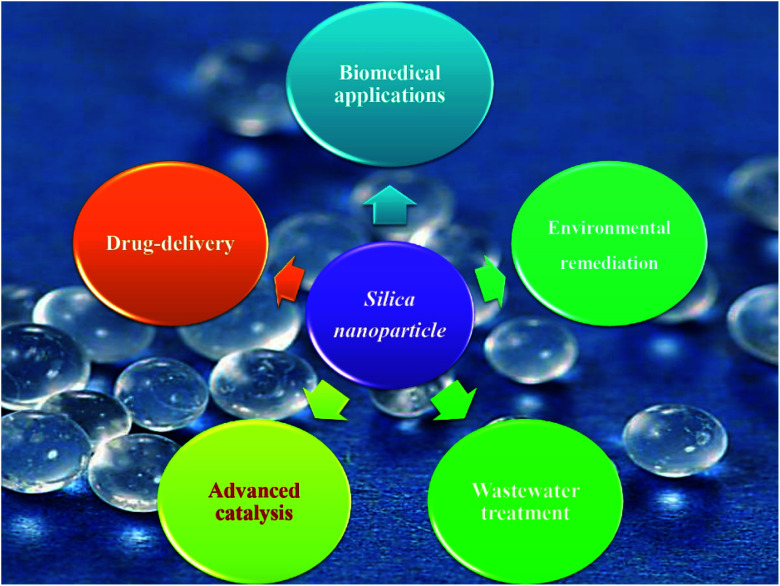
Graphical presentation for the major applications of nanosized silica.

Without a doubt, nanosized materials have attracted widespread attention due to their promising applications, where the outstanding features of these nanomaterials open the door for new types of technologies and applications.^14^ Therefore, the recent progress in nanomaterial technologies promises to overcome many challenges in the applications of advanced sciences. Silica nanomaterials have various applications such as advanced catalysis, drug delivery and biomedical applications, environmental remediation applications, and wastewater treatment^18^ (Fig. 2). Therefore, silica nanomaterials are considered one of the most used nanomaterials where the demand for this type of nanoparticles has increased dramatically to about 2.8 million metric tons in 2016 (ref. 19) with an annual growth of 5.6%.^20^

Chemical and biogenic synthesis are the most common techniques for the synthesis of silica nanoparticles. The chemical route has some undesirable characteristics: it produces some toxic compounds, is expensive, has high-energy consumptions, while the biogenic processes are cheaper, energy-saving, and eco-friendly for commercial-scale syntheses.^9,21^

**Fig. 3:**
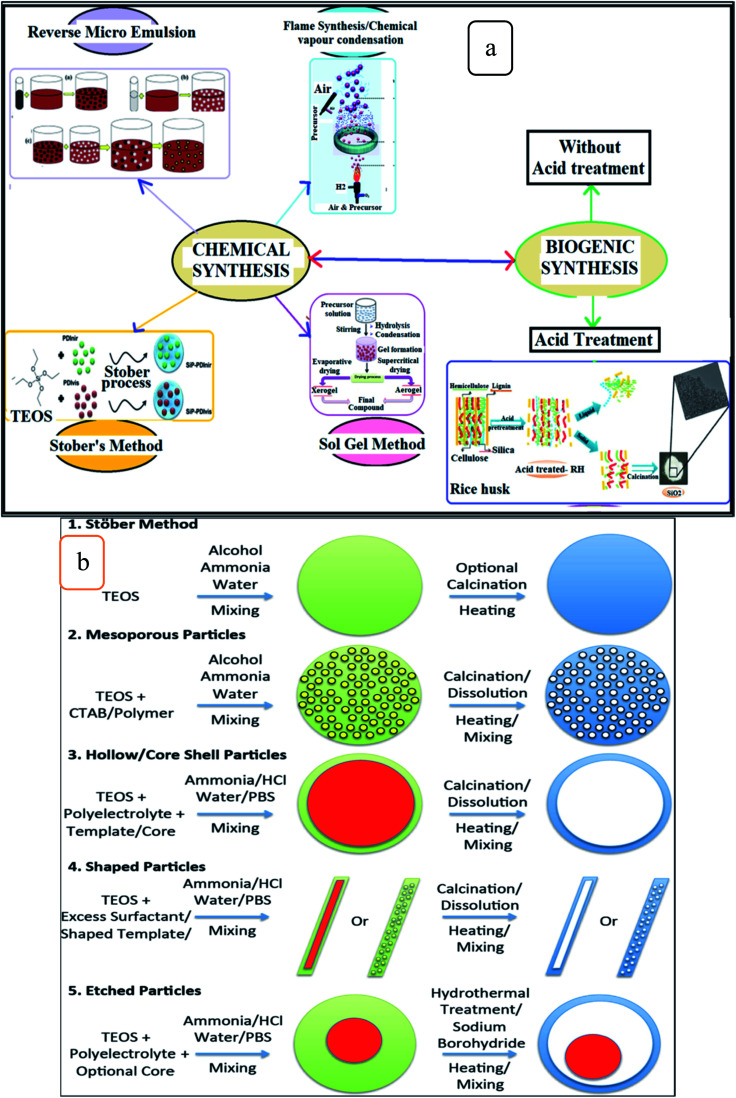
Illustration of (a) the chemical and biogenic synthesize of silica nanoparticles (this figure has been adapted/reproduced from ref. 9 with permission from Springer, copyright 2019), and (b) common methods used in the chemical synthesis of silica nanoparticles (this figure has been adapted/reproduced from ref. 21 with permission from Elsevier, copyright 2014).

Fig. 3 shows the chemical and biogenic synthesis of silica nanoparticles,^9^ as in Fig. 3a, and the most common techniques used in the chemical synthesis of silica nanoparticles are illustrated in Fig. 3b.^21^

Both the dimension and size of silica nanomaterials have intrinsic roles that govern their promising properties, where it was fabricated with one-, two-, and three-dimensional structures.^18^ Silica nanotubes, hollow silica-nanotubes, and silica nanofibers are other types of silica nanomaterials that attract great attention due to their novel properties, where it is extremely stable and less toxic. Thus, it has potential applications in different fields such as advanced catalysis, biomedical sciences, drug delivery, food technology, wastewater treatment, and energy storage/conversion.

Therefore, silica-based nanomaterials have various amazing features for their different applications.

### 2.1 Silica-based nanomaterials in advanced catalysis

During the last decades, a considerable number of novel solid catalysts have been employed to enhance the green synthesis of many chemicals.^14,22^ With an eye toward nanomaterials that are adequate to use in nanocatalysis, silica-based nanomaterials functionalized with different catalytic species have been reported in various fields. The core–shell structure of the catalysts with micro/mesoporous size distributions are widely applied in catalytic processes. Due to the promising properties of silica, such as chemical inertness, controlled porosity, and thermal stability, the metals nanoparticles coated by SiO_2_ become one of the most important catalysts of these types used in different fields.^23^

Recently, designing novel reactions, synthesis, and modification strategies in modern synthetic organic chemistry have gained great interest in both academic and industrial studies to improve the synthesis and fabrication of active materials with potential applications. Due to the specific physicochemical characteristics of various strategic materials such as platinum (Pt), cobalt (Co), copper (Cu), palladium (Pd), and gold (Au), they are extensively utilized in many modification and synthetic strategies in modern organic synthesis, especially in catalytic activities. The preparation of a novel bifunctional heterogeneous catalyst using mesoporous silica of type SBA-15 (Pt^2+^/SBA–APTE–SA) was investigated.^24^ The catalyst was completed with the functionalization of silica with (3-aminopropyl)triethoxysilane (APTES), succinic anhydride (SA), and a partial positively charged Pt^2+^. The prepared catalyst possesses superior catalytic activity toward the hydrosilylation of 1,1,1,3,5,5,5-heptamethyltrisiloxane (MDHM) with allyloxy polyethylene glycol (APEG) compared with a heterogeneous Pt-catalyst. Moreover, the prepared (Pt^2+^/SBA–APTE–SA) shows suitable activity in the hydrosilylation of other alkenes and shows good stability over five cycles. This catalyst shows future application as a green hydrosilylation process for the industry.^24^

**Fig. 4:**
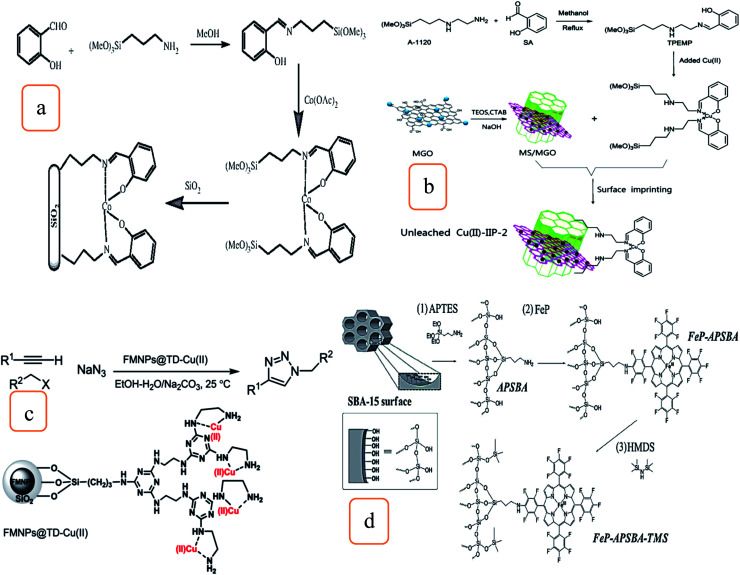
Schematic illustration of the modification strategy of (a) silica-supported Co^2+^ catalyst (this figure has been adapted/reproduced from ref. 25 with permission from Elsevier, copyright 2011), (b) preparation method of unleached Cu^2+^–IIP-2 (this figure has been adapted/reproduced from ref. 26 with permission from Elsevier, copyright 2020), (c) FMNP@TD–Cu^2+^ catalyzed one-pot preparation of 1,4-disubstituted 1,2,3-triazoles (this figure has been adapted/reproduced from ref. 27 with permission from the Royal Society of Chemistry, copyright 2016), and (d) (1) synthesis of the hybrid material APSBA from SBA-15; (2) FeP immobilization on the hybrid APSBA to prepare FeP–APSBA; and (3) silylation of the FeP–APSBA surfaces and HMDS to give FeP–APSBA–TMS (this figure has been adapted/reproduced from ref. 29 with permission from the Royal Society of Chemistry, copyright 2016).

In the presence of *N*-hydroxyphthalimide (NHPI), another approach demonstrated that Co^2+^–salen complex catalysts-modified silica was investigated and used for the aerobic oxidation of alkyl aromatics at atmospheric pressure shown in Fig. 4a. These reactions are selective for the oxidation of benzylic CH_2_, and ketone is the main yield. The catalyst can be efficiently employed for four cycles with the same catalytic activities.^25^

Fig. 4b shows the synthesis of a composite of mesoporous silica/magnetic graphene oxide composite (MSNs/MGO) as the support, which was modified by 2-((2-(3-(trimethoxysilyl)propylamino)ethylimino)methyl)phenol (TPEMP)-bearing Schiff base, surface Cu^2+^ ion-imprinted polymers (Cu^2+^–IIPs) were fabricated and used for the sorption of Cu^2+^ ions from aqueous media. The adsorption experiment can be repeated six times with the same efficiency. In addition, waste Cu(ii)-loaded adsorbent (MSNs/MGO–Cu) shows good catalytic performance to the manufacturing of 1-methyl-4-(*p*-tolyloxy)benzene with 95%.^26^

The click synthesis technique has been used to prepare the heterogeneous nanocatalyst *via* the immobilization of copper on magnetic silica nanoparticles modified with triazine dendrimer (FMNP@TD–Cu(ii)) (Fig. 4c). FMNP@TD–Cu(ii) succeeded in catalyzing the three-components reactions to synthesize 1,4-disubstituted 1,2,3-triazole from phenylacetylene compounds, sodium azide, and aromatic, heteroaromatic, allylic, and aliphatic halides. Catalytic reactions have good substrate scope, excellent yields, and selectivity. Moreover, the catalyst shows good stability and reusability.^27^

**Scheme 1:**
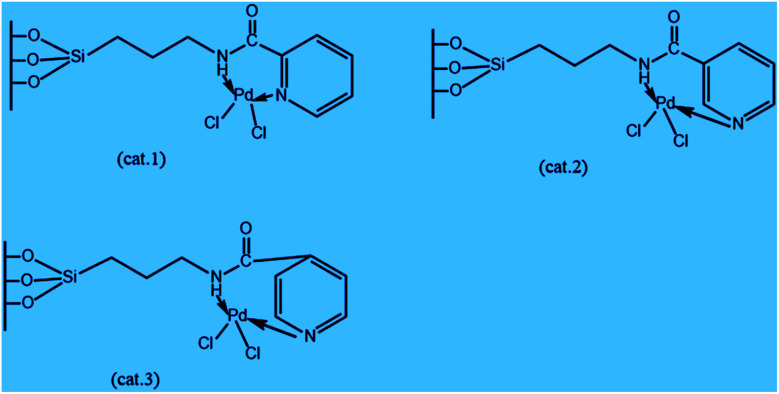
Schematic illustration structures of Pd-catalysts.^28^

A new catalyst was investigated by anchoring Pd(ii)–picolinamide complexes into mesoporous silica of type SBA-15 for the chemical transformation of cyclopentene. The characterization of the catalyst provides that the spatial differences of the complexes, particularly distances from –C=O to –N on the pyridyl cycle, may affect the electronic distributions of conjugated systems and, furthermore, the catalytic activities. The analytical results showed that cat.1 and cat.2 with 2-pyridinecarbonyl or 3-pyridinecarbonyl on the ligands produce better yield of cyclopentanone products, such as 96.2% conversion of cyclopentene and 76.3% yield of cyclopentanone in the presence of molecular oxygen as the sole oxidant, respectively. In addition, these catalysts appear to have the appealing ability of easy separation with recyclable features.^28^ The various structures of the fabricated Pd-catalysts are illustrated in Scheme 1.^28^

Two classes of catalysts were prepared by immobilizing iron porphyrins on mesoporous silica of SBA-type to oxidize hydrocarbons.^29^ These processes illustrated the crucial role of the silica surface to develop biomimetic catalysts and improve the activities. The first class was prepared *via* the immobilization 5,10,15,20-tetrakis(pentafluorophenyl)porphyrin iron(iii) chloride (FeP) on SBA-15 structures modified with (3-aminopropyl)triethoxysilane (APTES), designated as FeP–APSBA. The other class, FeP–APSBA–TMS catalysts, was prepared by the reactions of FeP–APSBA with 1,1,1,3,3,3-hexamethyldisilazane (HMDS). This will cause the partial replacement of sinol with non-polar Si–(CH_3_)_3_ (TMS) and yielded the FeP–APSBA–TMS catalyst, as shown in Fig. 4d.

The oxidation process of hydrocarbon gives information about the changes in the catalyst structure and its catalytic activity. It was reported that the sinol groups play a vital role in removing iodosylbenzene compounds, which act as oxygen donors in the oxidation reactions. It was shown that using the TMS end-capped group negatively affected the catalytic performance.^29^

**Fig. 5:**
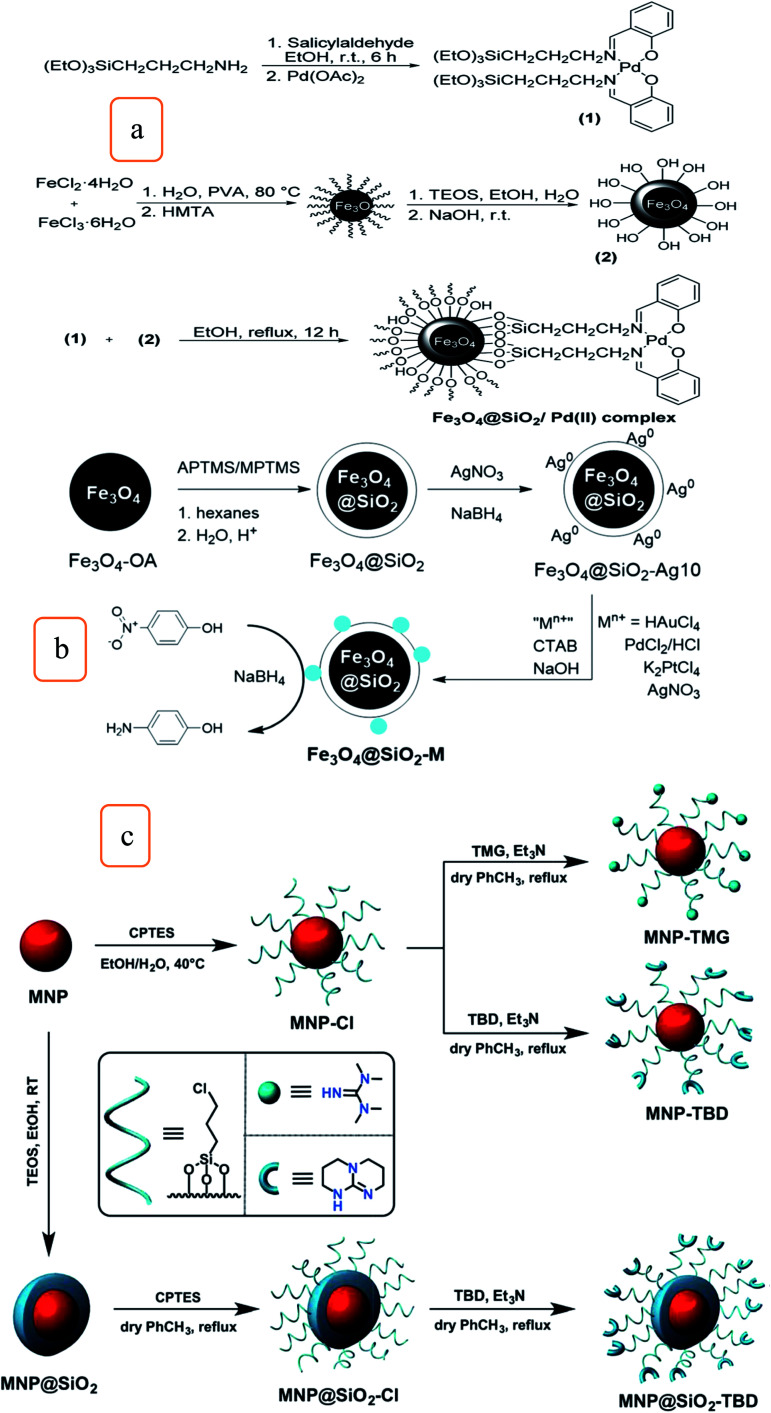
Schematic illustration for the synthesis of (a) Fe_3_O_4_@SiO_2_/Pd(ii) complexes (this figure has been adapted/reproduced from ref. 30 with permission from the Royal Society of Chemistry, copyright 2016), (b) procedure of Fe_3_O_4_@SiO_2_–M (M = Au, Pd, Ag, or PtAg), which were used to reduce 4-NP to 4-aminophenol (this figure has been adapted/reproduced from ref. 32 with permission from the Royal Society of Chemistry, copyright 2016), and (c) magnetic silica-coated and uncoated catalysts (MNP–TBD, MNP–TMG, and MNP@SiO_2_–TBD) (this figure has been adapted/reproduced from ref. 35 with permission from the Royal Society of Chemistry, copyright 2015).

A magnetic and recyclable composite Fe_3_O_4_@SiO_2_/Schiff base/Pd complex was synthesized for the fast and efficient *N*-arylation of carbamates, as shown in Fig. 5a. This catalyst was developed by an efficient, heterogeneous, and cost-effective method; the catalyst provides excellent yield, was easily recovered, and could be reused for six runs with the same activity.^30^

Recent nanotechnologies have led to novel nanomaterials, namely, magnetic nanoparticles (MNPs), that display simple surface functionalization and a high surface-to-volume ratio. These characteristics permit a high mass transfer rate and simple separation from the preparation media.

Simple removal using an external magnetic field yields promising immobilization supports for enzymes. Therefore, novel magnetic nanoparticles (MNPs) were fabricated by functionalization with EDTA–TMS. The fabricated materials were described using different analytical techniques such as TEM, FTIR, and BET. Then, the prepared magnetic nanoparticles were employed as the support for laccase immobilization to make favorable biocatalysts. It is known that EDTA–TMS has chelating characteristics. Therefore, it can be used to modify the surface of MNPs for laccase immobilization as a new strategy.^31^

On the other hand, the immobilized enzyme has favorable characteristics, and it was utilized for the biocatalytic degradation of some dyes. These fabricated magnetic nanoparticles with immobilized laccase exhibited significant advantages to use in industrial biochemical processes, biocatalysis, and biosensors.^31^

Also, the development of facile synthetic processes was investigated multifunctionalize a nanomaterial with the magnetic property of Fe-oxides with optical and catalytic properties of noble metals particles, which are considered to be a very significant object to realize the potential of hybrid nanoparticles.^32^ Therefore, the preparation of a catalyst composed of noble metals-decorated magnetic silica nanomaterials (Fe_3_O_4_@SiO_2_–M; M = Au, Pd, Ag, and PtAg) for the catalytic reduction of 4-nitrophenol was investigated and represented in Fig. 5b.^32^ It was found that the rate of the catalytic reduction activities related to the decorated nanoparticles are highest than that reported for nanosized particles, which are comparable and competitive to that of mesoparticles of similar composition.^32^ The data obtained shows promising magnetic response and its particles have appropriate magnetic recovery with >99% conversion for about four cycles. The particles of Fe_3_O_4_@SiO_2_–M prepared by this method demonstrate a considerable promise to further progress as a precursor to complicated anisotropic nanomaterials in various applications.

Recently and due to the unique ecofriendly properties of water, the use of water as a reaction media has gained considerable attention for many organic reactions, where the organic reaction in aqueous medium shows unique selectivity and reactivity for comparing with other media.^33^ Therefore, modifying silica-coated magnetic nanoparticles with phosphoric acid (g-Fe_2_O_3_@SiO_2_–PA) as recoverable, recyclable heterogeneous catalysts for the effective one-pot fabrication of α-amino phosphonates by Kabachnik–Fields reactions in water was reported.^33^ The synthesis of supported H_3_PO_4_ on magnetic silica nanoparticles (g-Fe_2_O_3_@SiO_2_–PA) was carried out for the preparation of a novel solid acid by reactions between chloro-functionalized g-Fe_2_O_3_@SiO_2_ and triethylphosphite, followed by hydrolysis. Phosphoric acid in g-Fe_2_O_3_@SiO_2_–PA provides an effective and appropriate acidic site, enhancing the prepared catalyst's catalytic activity. The catalyst showed a high yield and good reusable manner over five cycles.^33^

A magnetic silica nanocatalyst (Fe_3_O_4_@SiO_2_@PrSO_3_H) was synthesized by modification of (Fe_3_O_4_@SiO_2_) with a sulfonic group to form coumarin derivatives through the catalysis of the Pechmann condensation reaction of substituted phenols with ethyl acetoacetate. The catalyst was easily recovered and reused 22 times.^34^ Silica-coated and uncoated iron oxide nanoparticles were modified with two organic superbases, 1,5,7-triazabicyclo[4,4,0]dec-5-ene (TBD) and 1,1,3,3-tetramethylguanidine (TMG), to form three nanocatalysts composite (MNP–TBD, MNP–TMG, and MNP@SiO_2_–TBD) (Fig. 5c). The activities of nanocatalysts were evaluated in the methanolysis reaction of soybean oil under different conditions. It was found that MNP–TBD exhibits the highest yield (96%). It is important to note that the MNP@SiO_2_–TBD nanocatalyst composite's recyclability was preferable to that of MNP–TBD. This is because the silica coating prevents the dissolution of Fe_3_O_4_ nanoparticles by the free fatty acids in soybean oil.^35^

A thin mesoporous silica nanowire has been synthesized and integrates into three-dimensional (3D) networks.^36^ The fabricated networked nanowires were utilized to support MoO_2_ and examined for the catalysis and conversion of dibenzothiophene (C_12_H_8_S) to dibenzothiophene sulfone (C_12_H_8_O_2_S), as shown in Fig. 5c. The recyclability of the catalyst-adsorbent systems is explained.^36^

**Fig. 6:**
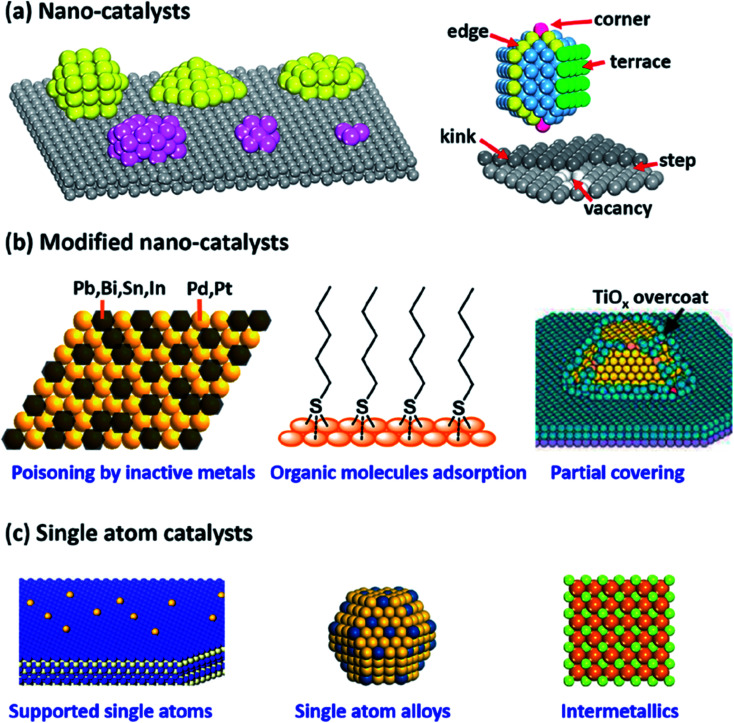
Shapes of various nanocatalysts; (a) supported nanocatalyst, (b) modified nanocatalyst, and (c) single-atom catalyst (this figure has been adapted/reproduced from ref. 37 with permission from American Chemical Society, copyright 2019).

The hydrogenation of carbonyl compounds is considered as an important technique used at a large scale in different industries such as petrochemicals, coal chemicals, fine chemicals, pharmaceuticals, and environmental industries.^37^ The efficiency of the hydrogenation processes depends on the selectivity and efficiency of the fabricated catalysts. Therefore, different generations of catalysts were developed and used, as reported in Fig. 6.^37^

Recently, various silica-based nanomaterials catalysts for the selective hydrogenation of different unsaturated organic compounds, such as acetylene, 1-hexyne, phenylacetylene, crotonaldehyde, 1-phenyl-1-propyne, furfural, cinnamaldehyde, nitroarenes, 3-nitrostyrene, and phenol have been fabricated and developed. For this purpose, various metals, such as Pt, Pd, Au, Ni, Ag, Fe, Ga, Rh, In, Mo, and Ru as well as bimetallic or alloys catalysts have been used and incorporated into to MSNs, as shown in Table 1.^38–57^

**Table 1:** Applications of silica-based nanomaterial catalysts in the hydrogenation of different unsaturated organic compounds

Catalyst	Substrate	Temperature (°C)	Pressure (MPa)	Conversion (%)	Selectivity (%)	Ref.
3D SiO_2_–NH–SO_3_H	Methylene chloride		0.5	96		38
SiO_2_@Ni/SiO_2_	Anillin	40	2	99		39
Au–Pd/SiO_2_	Acetylene	160	0.1	86	56.4	40
Au–Ni/SiO_2_	Acetylene	450		100	∼90%	41
PdAu–SAA/SiO_2_	1-Hexyne	25	0.5	100	85	42
Pd–La/SiO_2_	Acetylene	60	0.1	45	80	43
Ag/SiO_2_	Phenylacetylene	100	10	30	100	44
Ag–Pd/SiO_2_	Acetylene	160	0.1	93	80	45
30.8% HPW@NPM-SiO_2_	*n*-Octane	90	3	99		46
Pd–Ag/SiO_2_	Acrolein	200	5		31	47
Ag–Ni/SiO_2_	Acetylene	160	0.1	90.4	31.4	48
Ni–In/SiO_2_	Acetylene	180	0.1	63	100	49
Ni_5_ Ga/SiO_2_	Acetylene	180	0.1	100	81	50
Pt/m-SiO_2_	Ethyl pyruvate		5	90	82	51
Fe(NiFe)O_4_–SiO_2_	Furfural	90	20	94.3	∼100	52
Pt–FeO/SiO_2_	Cinnamaldehyde	25	1	99	87	53
Rh–In/SiO_2_	Nitroarenes	75	1	99	91	54
Ru–Mo/SiO_2_	3-Nitrostyrene	30	3	97	98	55
NiCo/Si–Ti	Phenol	100	1	98.2	>99	56
Rh@HMSNs	Phenol	45	0.5	100	97.7	57

Table 1 shows the effect of the fabricated catalysts on the hydrogenation of some unsaturated compounds in both the gas and liquid phases at optimum temperature and pressure conditions with recorded conversion and selectivity percentages. The results reported show that it is difficult for some metals/metal oxides to cleave with H_2_, and therefore, extremely low hydride covering on the metals/metal oxides surface participates in the prevention of over-hydrogenation side-reactions.^37^

For the catalytic reduction processes of nitroaromatics, *e.g.*, 4-nitrophenol (4-NP), 2-nitrophenol (2-NP), and 2-nitroaniline (2-NA), bimetallic Ag–Ni@SBA–16C nanocatalysts were fabricated and tested.^54^ The data obtained showed especially high catalytic activities to 4-NP, 2-NP, and 2-NA reduction comparing with monometallic counterparts Ag/Ni with SBA–16C. Also, these types of nanocatalysts have a highly active character and can be facilely recovered.

Therefore, Ag–Ni@SBA–16C nanocatalysts are considered as promising nanocatalysts for heterogeneous catalysis in reducing nitroaromatic compounds.^58^

**Fig. 7:**
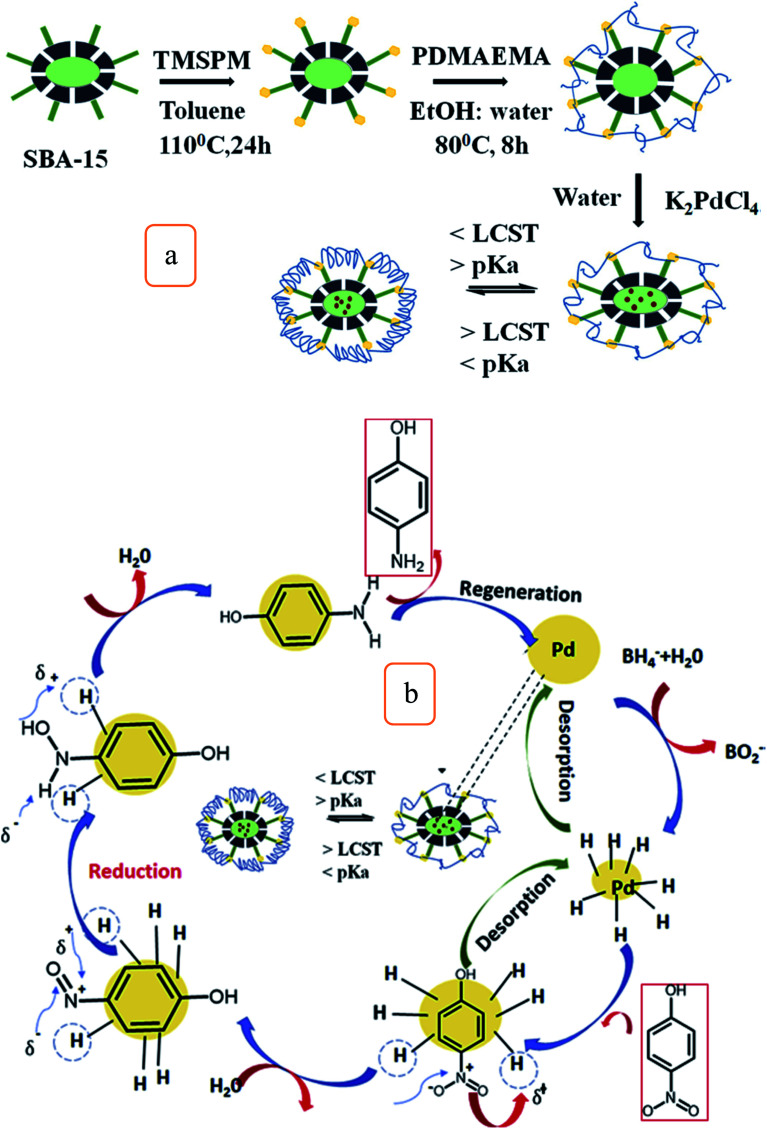
(a) Schematic illustration of the fabrication of Pd/m-SBA-15/PDMAEMA hybrid catalyst, and (b) recyclability of the hybrid catalysts on the reduction of 4-NP (this figure has been adapted/reproduced from ref. 59 with permission from Elsevier, copyright 2019).

Also, a novel Pd/m-SBA-15/PDMAEMA catalyst was fabricated by the free radical polymerization of [2-(*N*,*N*-dimethylamino)ethyl-methacrylate] (DMAEMA). Pd nanoparticles were incorporated with the polymerized m-SBA-15 to form Pd/m-SBA 15/PDMAEMA novel catalyst (Fig. 7a), which shows considerable catalytic activities to reduce 4-nitrophenol (4-NP) into 4-aminophenol (4-AP) at lower pH and temperatures. Pd/m-SBA 15/PDMAEMA exhibited considerable reusability for about 4 cycles without any substantial loss of its activities (Fig. 7b) and the results showed that this novel catalyst can have promising applications in the bio-medicine field.^59^ Also, another type of carboxylate catalysts of type MIL-101(Fe)/SiO_2_, the Material Institute Lavoisier (MIL), has been fabricated and tested to enhance the catalytic activities of reduced nitroaromatic compounds.^60^ This catalyst demonstrates excellent catalytic activity to reduce *p*-nitrophenol with a considerable conversion of 93.8% in 4 min. These special activities can be due to the interfacial effects between SiO_2_ and MIL-101(Fe) complexes.^60^

A facile green chemical reduction process is investigated to develop Pd/RGO nanocomposite materials by exfoliated graphite oxide as a precursor in the presence of different reducing agents. In these nanocomposite materials, Pd nanoparticles are dispersed on graphene surfaces. The catalytic activities of the prepared nanocomposite have been tested to reduce nitrobenzene in the presence of some reducing agents. The results show that the catalytic activities and performance of synthetic Pd/RGO nanocomposite material catalysts depend on the concentrations of targets, catalyst loading, time of reactions, and the nature of Pd nanoparticles.^61^

The preparation of two composite structures Fe_3_O_4_/SiO_2_/Ag nanocubes and SiO_2_/Ag nanospheres for the catalytic reduction of 4-nitroaniline to 4-phenylenediamine was developed.^62^ It was noted that the as-synthesized Fe_3_O_4_/SiO_2_/Ag nanocubes exhibit high catalytic reduction efficiency of 4-nitroaniline to 4-phenylenediamine compared to Fe_3_O_4_ nanocubes, Ag, and SiO_2_/Ag nanoparticles. Moreover, silver-grafted catalyst was used for fifteen runs with the same catalytic efficiency.^62^

**Fig. 8:**
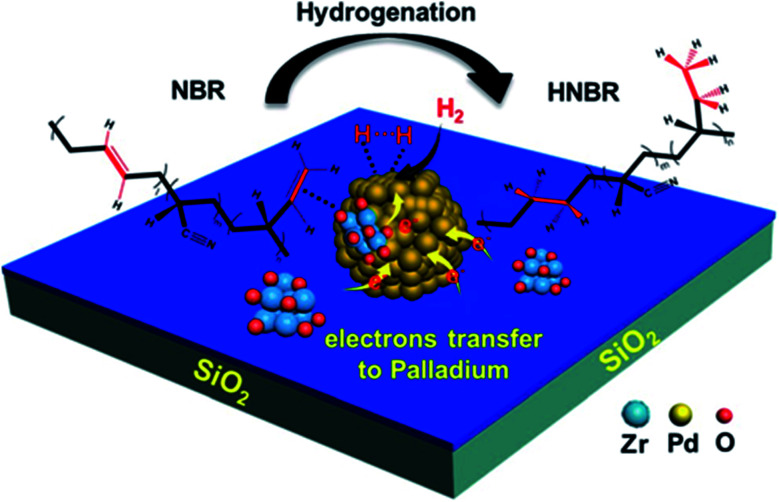
Catalytic procedure for the hydrogenation of NBR to HNBR (this figure has been adapted/reproduced from ref. 63 with permission from Elsevier, copyright 2020).

Due to the large pores of SiO_2_, it is chosen as a support nanomaterial for some catalysts. It is used to modify some functional groups, such as the amino groups and the hydrogenation of nitrile butadiene rubber (NBR) to HNBR, utilizing three PdZr bi-component nanocatalysts supported on modified silica, as shown in Fig. 8.^63^ In the hydrogenation processes, both NBR and H_2_ molecules are adsorbed together on the surface of Pd nanoparticles. In the first step, hydrogen molecules (H_2_) are disassociated into two hydride species (Pd–H); in the second step, the hydrogen atoms attack the double bonds in C=C in the adsorbed nitrile butadiene rubber (NBR) molecules. Due to the transfer of electrons from both Zr and N–SiO_2_ species to the particles of Pd, the electron-rich Pd active sites in the PdZr bi-component catalysts lead to a decrease in the reaction barriers to promote the catalytic activities.^63^

### 2.2 Silica-based nanomaterial in drug-delivery and biomedical applications

Nowadays, the development of unique nanomaterials has received considerable attention in biomedical technologies, *e.g.*, drug delivery, gene transfections, cancer diagnosis, and therapy,^64^ Numerous therapeutic methods suffer from stability issues, failure to pass through the membrane of cells, and fast clearance *in vivo*. Therefore, nanomaterials can solve these issues and provide potent tools for delivering and releasing drugs, where the biosafety of these nanoparticles is considered as an important requirement for the use of NPs in drug delivery systems.^65,66^

Due to the promising advantages of MSNs, such as unique morphology, geometry, and the ability of the nanoparticles of fine delivery and target particular organs/tissue, have gained great attention in biological and medical fields as carriers for different therapeutic agents. The large pores of MSNs act as reservoirs to carry therapeutic drugs and surface functionalization to allow adequate control of drug release, and its biocompatibility can also be enhanced by modifying its surface.^9,67,68^ Also, due to the mesoporous structures of MSNs, the drug can be homogeneously distributed through its matrix systems compared to other drug delivery systems.^69^ Therefore, many works have been achieved to functionalize drug-loaded MSNs by targeting ligands and stimuli-sensitive materials, where anti-cancer drugs can be delivered to their targets by MSNs, which respond to multiple stimuli.^68^ Also, MSNs are considered as a promising carrier for gene delivery. Thus, various strategies are developed to fabricate specific multifunctional nanocarriers; thus, a novel technique for drug design based on nanomaterials and related nanostructures for operative delivery drugs is of distinguished significance in future medical treatment.^66^

On comparing MSNs with other drug delivery systems, it is easily observed that the mesoporous structures of MSNs provide homogeneous distributions of drugs through the matrix systems and have higher drug loading capacities. Moreover, it does not prompt the hemolysis of RBCs and is not genotoxic. Therefore, they are considered as biologically safe carriers to deliver drugs or genes.^70^

Recently, many works have been reported and developed for cancer treatment. There are still many challenges in cancer treatment, such as the affected site or organ in the body, side effects to the non-diseased cell, and drug delivery to cancer cells.^68^ For example, the treatments of brain and pancreas cancer cells by removing through surgery, chemotherapies, and radiotherapies are complicated due to their sites and the difficulty of crossing of the drugs through the blood–brain barrier to the brain cells and the positions of the pancreas close to the liver and intestine. Also, due to the different limitations of traditional therapies and drug delivery systems (DDS), new strategies to deliver drugs at the target sites are developed. Also, MSNs have been proved to be promising nanomaterials that aid in developing drug-delivery systems applications and siRNA delivery, where it can successfully deliver siRNA inside the cells to enable gene silencing and co-deliveries with another molecule, and can possibly show synergistic therapeutic effects.

**Fig. 9:**
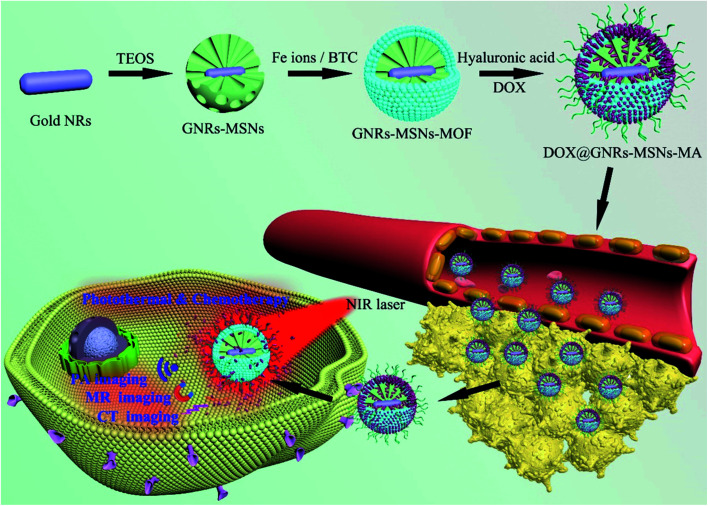
Synthetic procedure of GNRs–MSNs–MA for targeted chemo–photothermal combination therapy and tri-modal MR/CT/PA imaging (this figure has been adapted/reproduced from ref. 73 with permission from Elsevier, copyright 2020).

Azizi *et al.*^69^ synthesized a novel composite formed from the functionalization of folic acid (FA) to Tb@KCC-1–NH_2_–FA (terbium-doped dendritic fibrous) nanocomposite that has a highly mesoporous surface area by a novel hydrothermal protocol. This composite has FA moieties on its surface and, therefore, can easily interact with folate receptors (FR) on the surface of the different cancer cells. The cytotoxicity of this synthesized composite was tested for HT 29 colon cancer, MDA breast cancer, and HEK 293 Normal cells. The results confirm that this composite can be used as a nontoxic catalyst or carrier in a biological ambience.^69^ Another type of MSNs functionalized by the polyamino ligands *N*1-(3-trimethoxysilylpropyl)diethylenetriamine to produce the composite MSN–DETATMS (M1) nanomaterials, which then reacts with diphenyl tin(iv) to form MSN–DETATMS–O_2_–SnPh_2_ (M2), which was modified by FA to obtain the combination of the folate fragments from the formation of an amido bond [MSNDETATMS–FA] (M3) and then reacted with Sn1 to form [tin-functionalized material] (MSN DETATMS–FA–O_2_–SnPh_2_) (M4). These nanocomposites were synthesized and characterized with and without a folate fragment in their structures and then examined *in vitro versus* various cancer and non-cancer cells to detect the cytotoxic mechanisms.^71^ The cytotoxic tests showed clear relationships between the cytotoxicity and accumulation of the composite inside the cell. The results demonstrate that the cell uptakes are considerably higher for those nanoparticles with FA fragments in cells than that of fast folate receptors such as MDA-MB-231 and A549. This is evidence that the folate strategies to enhance cell internalizations are sufficient to improve the potential cytotoxic nature of the particles. This study concludes that tin-containing MSNs functionalized with folate fragments (M4) have potential applicability in future chemotherapeutic treatments.^71^ Recently, metal–organic framework (MOF) was deposited on core–shell Au-nanorods [GNRs]–mesoporous-silica nanoparticles MSNs to form GNRs–MSNs-MOF and then modified with hyaluronic acid (HA) to fabricate highly efficient GNRs–MSNs–MA, as in Fig. 9, integrate targeted chemo–photothermal combination therapy and tri-modals (MR) magnetic resonance, (CT) computed tomography, and (PA) photoacoustic imaging into a single platform.^72^ GNRs–MSNs–MA exhibits excellent photothermal effects and improves the drug loading efficiency and laser-triggered drug release with precise and efficient targeted abilities toward cancer cells.^73^

Thorat *et al.*^74^ studied the development of supra-assembled silica-nanocargo, which enhances anti-cancer drugs for targeted chemotherapeutics cargo delivery to overcome chemoresistance in cancer cells *in vitro* and *in vivo*. They accomplished that the encapsulation of different anti-cancer moieties in a single nanomedicine modality delivered the drug to cancer cells with considerable loading and release, and overcame the barrier of targeting the drug-resistance cancer cells. The triple-action of the supra-assembled silica-nanocargo lead to the efficacious killing of drug-resistance colon cancer cells *in vitro* (∼92% cell death).^74^ Moreover, *in vivo* animal toxicity examinations showed that supra-assembled nanocargos have encouraging safety index to be utilized in cancer treatment and drug-delivery applications.^74^

For bone drug delivery systems *in vitro*, amino-modified mesoporous silica SBA-15 pellets as bifunctional composite-type pellets based on cefazolin-SBA-15 and microwave-assisted hydroxyapatite was used as a proposed drug delivery systems to avoid prolonged drug release and mineralization potential.^75^ Prolonged (5 days) release of cefazolin from the prepared pellets and exhibiting mineralization potential in simulated body fluid (after 28 days of incubation) were observed. The proposed pellets have no cytotoxic effects of pellets toward human osteoblasts.^75^ Also, new chlorine photosensitizer pyropheophorbide-a (PPA) were functionalized with Fe_3_O_4_@SiO_2_@CS nanoparticles to prepare fluorescent nanoparticles Fe_3_O_4_@SiO_2_@CS@PPA (MFCSPPA) for photodynamic therapy. In addition, the results in this work showed that in the presence of fluorescence detectors (such as 1,3-diphenylisobenzofuran (DPBF)), MFCSPPA mediated singlet oxygen production in solution conditions. Moreover, these results explained that after treatment with PDT, two types of compatible mechanisms might play a vital role in producing reactive oxygen species in HeLa cells. This research proved the possibilities of preparing various magnetic fluorescence photosensitizers, Fe_3_O_4_@SiO_2_@CS@PPA, which is expected to be a novel candidate of the PDT agent and medical imaging agent, and has different applications in simultaneous applications medical fluorescence imaging, biological probes, MRI, and magnetic hyperthermia.^76^

Table 2 represents various silica-based nanomaterials and their applications in some types of cancer therapy.^77–100^

**Table 2:** Applications of silica-based nanomaterials in cancer therapy^77–100^

Type of cancer	Drug type	Type of targeted cells	Functionalized material	Ref.
Bone	Doxorubicin (DOX)	MC3T3-E1 preosteoblastic cell line	PAA-capped MSNs	77
	Alendronate (AL)	MG-63	Mesoporous bioactive glass (MBG)	78
	DOX	L-02 and SK-BR-3 cells	Poly(tannic acid) modified MSNs	79
	DOX	L-929	Biodegradable hollow mesoporous-silica nanoparticles (dHMLB)	80
Breast	DOX	MCF-7 and MDA-MB-231 cancer cells lines	DOX–MSN–SS–CH–FA	81
	Anastrozole (ATZ)	MCF-7 cancer cells	MSN–ATZ–CH–FA	82
	Umbelliferone	MCF-7 and MCF-10a	Umbe@MSN–PAA–FA	83
	DOX	MCF-7 and KB cells lines	bMSN-ss-COOH	84
	Paclitaxel and curcumin	Canine breast cancer cell line	Lipid bilayer-coated with MSNs	85
	DOX	MCF-7 cell line	MSN-coated with Au-nanorods	86
Brain	DOX	U-87 MG-luc2 cells	Tf-functionalized MSNs	87
	DOX	U87 glioma cells	MSNs modified with arginylglycylaspartic acid peptide	88
Colon	CytC AS1411 DOX	HCT116 cells	Nanoporous structure of porous silica nanocargo	89
	Oxaliplatin (OXL) and miRNA-204-5p	HT-29 cells	HSMN	90
	5-Fluorouracil (5-FU)	HT-29	MSN–P(OEGMA-*co*-RGD)	91
Liver	ATO	SC-7721	LPMSNs	92
	Sorafenib	HCC	Gold nanoshell MSNs	93
Lung	Plasmid and CRISPR ribonucleoprotein	A549 and HeLa cervical	Lipid-coated mesoporous silica nanoparticle (LC-MSN)	94
	Bortezomib	A549 cells and H1299 cells	HMSNs	95
	Cisplatin	A549, A2780 and MCF-7	Carboxyl group-functionalised MSNs (COOHMSNs)	96
	Myricetin (Myr)	Non-small cell lung cancer (NSCLC)	MSNs conjugated with MRP-1 siRNA and FA	97
	Doxorubicin (DOX)	A549 cancer cells	MSN	98
Prostate	DOX	LNCaP-AI	MSN@CaCO_3_@CM	99
	DOX	LNCaP-AI	PMSA surface-modified MSN	100

Other works proposed a drug-delivery system of cisplatin consisting of magnetic silica nanoparticles modified with (3-aminopropyl)-trimethoxysilane (APTS), as a coupling agent of cisplatin, in which the nanoparticles were coated with chitosan to improve their biocompatibility and control the drug release.^101^ Song *et al.*^102^ investigated the use of MSNs-based DNA vaccine, exhibiting numerous antibody productions and strong T-cell activation mediated by Ram–MSNs–PEI. MSNs enabled effective antigen expression intracellularly and concurrently activated the maturation of the dendritic cells with promoted CD80 and CD86 expressions. Promoted antigen-specific IgG productions, elevated cytokine secretion of IFN-γ, and enhanced population of CD8 T cells were obtained for the SNs-mediated DNA vaccine, exhibiting excellent performance compared to the other materials. This Ram–MSNs–PEI-based formalization provides essential insights for the rational design of operative nanotechnologies-enabled DNA vaccine, potentially inspiring future research for diseases treatment such as chronic infections and cancer.^102^ Functional mesoporous silica nanoparticles (F-MSNs) with levorotatory (FL-MSNs) and dextrorotatory (FD-MSNs) forms have been synthesized and characterized to examine their specific advantages in delivering drugs molecules using nimesulide (NMS) as a model drug.^103^ The data obtained represents the preparation of both FL-MSNs and FD-MSNs by the co-condensation technique *via* chiral molecular silica coupling agents. *In vivo* pharmacokinetics and anti-inflammatory pharmacodynamics data showed that both FL-MSNs and FDMSNs enhance the oral bioavailability of NMS (698.45% and 887.03%, respectively). FD-MSNs delivered more NMS after making a response to the *in vivo* environment. They then showed considerable anti-inflammatory pharmacodynamics efficacy.^103^

In the drug delivery systems, several types of SNPs-based materials loaded with different drugs and various works have been published in this field. Although there are several advantages of SNPs-based materials drug-loading processes, not every drug can be loaded with high concentrations, thus increasing the doses of SNPs-based materials required to attain good therapeutic effects.^104^ Fluorescence labeling and internal cavity magnetic loading of the shell of SNPs authorize therapeutic tracking of the nanocapsule using fluorescence and electron microscopy. Thus, silica nanocapsule illustrates promising theranostic nanoplatforms for targeted drug delivery systems.^105^ Therefore, it is important to continue the experimental studies to develop the functions of SNPs-based materials, especially to prolong its circulation *in vivo* and the human tolerance of its unloaded MSNs, to enhance its performance and likely clinical advancement for cancer treatment.^104^ Due to these, all amazing properties of mesoporous silica-based nanomaterials play a vital role in combinatorial photo-chemotherapy such as photodynamic-chemotherapy, photothermal-chemotherapy, and photodynamic–photothermal-chemotherapy.^106^ Core–shell structured nanohybrid (MSNs@carbon dot (C-dot)/RB/DOX) was fabricated and tested for bioimaging-directed photodynamic-chemo-therapy.^107^ Multifunctional I/D@PEG–MSNs–Pep with a photothermal-activated gatekeeper (Azo-CD) nanostructures were fabricated to efficiently combine cancer therapeutics.^108^ Also, redox-sensitive PEGylated DOX@MSNs–BDP–PEG nanomaterials were fabricated to reduce solid tumors by integrating NIRF boron dipyrromethene (BODIPY)-associated hyperthermia under 638 nm laser irradiation with DOX-induced chemotherapy.^109^ Quantum dots have been considered as one of the significant nanoprobes utilized in imaging.^9^ Due to the photosensitive properties of carbon quantum dots (CQDs) and their ability to provide singlet oxygen, they are considered new in cancer tumor imaging and treatment. Therefore, pH/redox/enzyme responsive SNPs carriers with carbon quantum dots were fabricated to use in photodynamic-chemotherapy.^110^ Another new core–interlayer–shell DOX/ZnPc co-loaded MSNs@pH-sensitive CaP@PEGylated liposome was fabricated using mesoporous silica hybrids (MSNs@CaP@PEGylated liposomes) and extruding DOX-loaded MSNs@CaP into ZnPc-loaded PEGylated liposomes for collaborative PDT and to improve synergetic chemo-photodynamic therapy.^111^ DOX-loaded MSNs-coated Au-cube-in-cubes nanocomplex (RGD-CCmMC/DOX) was constructed with RGD peptide modifications as a triple mode of combinatorial therapy based on mesoporous silica for multimodal imaging and tumor-targeted triple-synergetic cancer-therapy.^112^ Self-decomposable mDOX@SiO_2_/MB was synthesized to overcome the disadvantages of conventional MSNs, such as potential cytotoxicities of organosilane precursors and hemolysis, for nuclear-targeted PDT and chemotherapy.^113^ Also, silica nanoparticles have great attention and have been strongly utilized in other biomedical fields due to their favorable characteristics and great development potential.^114,115^ Therefore, silica nanoparticles show different features to construct MSN-incorporated hybrid tissue-engineering scaffolds and specific induced activities toward stem cells.^115^ An operative and simple process was developed to use a suspension of MSNs to recover latent blood fingermarks on dark surfaces at crime scenes. This method utilizes the photonic crystal effect of monodisperse silica nanoparticles to improve latent blood fingermarks kept on black plastic bags surfaces for at least 30 days without the requirement of surfaces to functionalize and conjugate the dye or fluorescently label the molecules.^116^ It has been widely known that the biodegradability of conventional silica nanoparticles is limited. Hence, the progression of intrinsically biodegradable SNPs has great attention and has aroused extensive research interest during the last decade, and many strategies have been investigated to enhance the biodegradability. Although the promising properties of functionalized silica nanoparticles (SiO_2_ NPs) as a biosafe delivery platform for drugs and genes have been established, their abilities as a carrier of target cancer cells may be developed more by decorating its solid surface with antibody fragments or peptides.^70^

### 2.3 Silica-based nanomaterials in environmental remediation applications and wastewater treatment

During the last decade, water scarcity in arid environments has increased due to environmental and water pollution, which are becoming a major issue facing all societies today, resulting in environmental retardation and very dangerous human and animal health issues.^117,118^ Therefore, different types of ecofriendly nanomaterials have been synthesized and fabricated to decontaminate and treat such types of wastes. A new composite of magnetic (Dy_*x*_MnFe_2−*x*_O_4_) nanoparticles decorated on mesoporous silica particles was fabricated and removed from different organic pollutants. This synthetic composite's efficient removal, eco-friendly, and economical catalytic properties are due to its mesoporous characteristics and the remaining dyes contents were degraded by the photocatalytic activities of Dy_*x*_MnFe_2−*x*_O_4_ nanoparticles on them.^118^ This investigated composite has a considerable synergistic effect on the photocatalytic activities and adsorption of environmental waste treatment.^118^

**Fig. 10:**
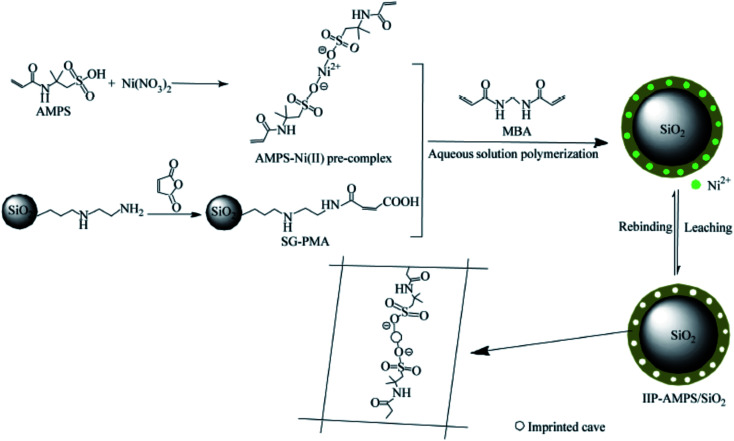
Synthetic procedure for IIP–AMPS/SiO_2_ (this figure has been adapted/reproduced from ref. 119 with permission from Chinese Chemical Society, Institute of Chemistry, Chinese Academy of Sciences and Springer-Verlag GmbH Germany, part of Springer Nature, copyright 2018).

Another novel Ni^2+^ ion-imprinted probe was synthesized from silica gel polymer combined with medium aqueous polymerization with 2-acrylamido-2-methyl-1-propanesulfonic acid (AMPS) as a functional monomer for the selective recovery of Ni^2+^ from aqueous media, as in Fig. 10. The maximum removal capacity of the ion-imprinted composites to Ni^2+^ was 66.22 mg g^−1^ at a pH of 7.0. The investigated novel composite's relative selectivity coefficients of Ni^2+^/Co^2+^, Ni^2+^/Cu^2+^, Ni^2+^/Zn^2+^, and Ni^2+^/Pb^2+^ were 9.23, 15.71, 14.72, and 20.15, respectively. The composite shows good regeneration properties.^119^

**Fig. 11:**
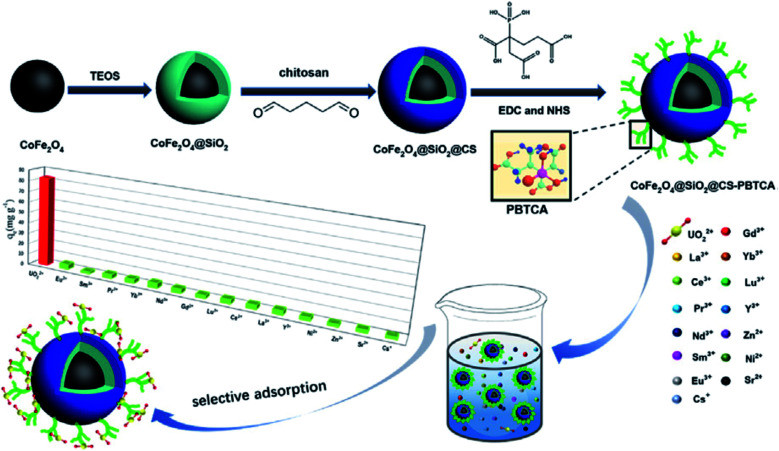
Fabrication of sorbent, 2-phosphonobutane-1,2,4-tricarboxylic acid (PBTCA)-decorated chitosan-coated magnetic silica nanoparticles (this figure has been adapted/reproduced from ref. 120 with permission from Elsevier, copyright 2020).

The decoration of chitosan-coated magnetic silica nanoparticles with 2-phosphonobutane-1,2,4-tricarboxylic acid (PBTCA) (CoFe_2_O_4_@SiO_2_@CS-PBTCA) for the selective removal of uranium from aqueous solution was investigated and discussed (Fig. 11).^120^ Even in the presence of 14 coexisting cations, the composite can select uranium adsorption with the adsorption capacity reaching 83.16 mg g^−1^, which was considerably higher than that of ungrafted CoFe_2_O_4_@SiO_2_@CS (29.99 mg g^−1^). Also, the presence of silica prevents the dissolution of iron and cobalt in acidic solutions. The results obtained from X-ray photoelectron and FTIR spectroscopy showed that the carboxylic groups and phosphonic groups in PBTCA were responsible for U(vi) capture.^120^

A highly effective method to remove Pb^2+^ from acidic wastewater was studied by introducing Pb^2+^-imprinted mesoporous silica (PbIMSNs) SBA-15 by combining co-condensation and functionalized iminodiacetic acid (IDA). Moreover, XPS analysis provided that Pb^2+^ ions were bonded by the N-atom and carboxyl O-atom of iminodiacetic acid. The composite shows the selective adsorption of Pb^2+^ competing with six metals. The adsorption kinetics reached equilibrium in 7 min, and the adsorption efficiency reached 93% after six runs.^121^ Various works reported the vital role of silica and its nanomaterials in the water treatment and removal processes of different impurities; therefore, it has gained great attention due to its potential adsorption and/or catalytic applications.^122^ 1-*n*-Propyl-3-methylimidazolium chloride modified silica for the adsorption of Co^2+^, Cu^2+^, Zn^2+^, Cd^2+^, and Hg^2+^ chlorides from alcoholic and aqueous media were investigated, where the sorption mechanism proceeded by the formation of metal chloride anionic complexes inside near the grafted organic cations as counter ions.^123^ The results obtained are confirmed by different types of analytical methods. Higher adsorption percentages of the investigated metal–chloride complexes from aqueous media were obtained. The suitable models of sorption equilibrium explain the dissociation and auto-complexation of chloride ions in media and form anions of multi-chlorometallate in the solid phase. In addition, the equilibrium constants for the sorption of CoCl_2_ and CuCl_2_ using various materials containing grafted organic cations and chloride counter-ions were reevaluated in the framework. The results obtained proved that the equilibrium constant of these processes is independent of immobilized cations and carriers.^123^

An iminodiacetic acid chelator's modified magnetic silica nanoparticles (FS@IDA) were developed to complex heavy metals in soil. The experimental results demonstrated that Cd^2+^, Zn^2+^, Pb^2+^, Cu^2+^, and Ni^2+^ carbonates, PbSO_4_, and PbCl_2_ in water-insoluble salt systems show that FS@IDA could efficiently chelate with these heavy metals. The resulting chelates of FS@IDA–Cd and FS@IDA–Pb are separated by magnetic methods, resulting in the adsorption of 84.9% and 72.2% for Cd^2+^ and Pb^2+^, respectively. Also, FS@IDA adsorb heavy metals bound to the organic matter in the soil sample.^124^

Magnetic silica nanoparticles-coated with FeCo/graphitic carbon shell nanocrystals (FeCo/GC NCs@MSNs) to interact with heavy metals and organic dyes were fabricated by the thermal decomposition of metal precursors in MSNs and subsequent methane CVD. The graphitic carbon protects the nanoparticles from oxidation and acid leaching in concentrated hydrochloric acid and cooperative non-covalent, hydrophobic interactions with organic dyes. Further functionalization of the surface of FeCo/GC NCs@MSNs with thiol groups enables the nanoparticles to bond with Hg^2+^ ions and organic dyes.^125^ Novel nanocopper–ferrocyanide–silica (SiO_2_–CuFe–CN) NPs were prepared and used as promising nanoparticles with the highly selective removal of ^99^Mo radioisotope from radioactive waste solutions from the solutions of ^134^Cs, ^60^Co, and ^90^Sr radioisotopes.^126^

Hollow-sphere silica nanoparticles modified with βCD-SNHS were successfully investigated as a novel sorbent for methylene blue (MB) from aqueous media. The functionalization process enhances the adsorption performance of the nanoparticles. This is because βCD has hydroxyl groups and the inner cores of the hydrophobic cavity (host–guest interactions), which can form complexes with organic pollutants.^127^ Hg(ii) adsorption on immobilized Mn silica-based nanoparticles was investigated.^128^ Electron paramagnetic resonance (EPR), cyclic voltammetry (CV), and electrochemical impedance spectroscopy (EIS) of manganese ions proved that most of the covalently bound active sites of the nanoadsorbents are in the form of Mn^3+^ ions at the surface. Hg(ii) adsorption diminished the electron transfer rate of the reactions, referred to as Mn^3+^/Mn^4+^ redox couples. The maximum sorption capacity is 289.5 mg g^−1^ and the composite can be recycled for 7 cycles.^128^

Thiourea-modified silica gel (SG-Pr-THIO) was prepared for adsorption and catalysis purpose. The organo-functionalized surface showed adsorption ability toward the metal ions Cd^2+^, Cu^2+^, Ni^2+^, Pb^2+^, and Co^2+^ from some aqueous media. SG-Pr-THIO was bonded with Mo^2+^ to form an organometallic complex for catalytic applications, forming a novel composite material SG-Pr-THIO-Mo, which can be a catalyst in the epoxidation of cyclooctene and styrene.^129^ Also, the modification of mesoporous silica with hydroxyphosphate ethyl pendant groups (POH-MS) was investigated to remove lead ions (Pb^2+^) from aqueous media. The modified nanoparticles showed high efficiency of hydroxyphosphate ethyl groups to Pb^2+^ with a maximum adsorption capacity of 272 mg g^−1^.^130^

A novel green nanocomposite of nanosilica (SiO_2_@VB9) was formed by surface immobilization and chemical bonding of folic acid (VB9) as ecofriendly chemical compounds and nanosilica (nano SiO_2_) in the chloride form and was examined for the removal and preconcentration of Cu^2+^, Pb^2+^, and Cd^2+^ ions from water.^131^ This investigated composite shows perfect removal capacities of these toxic heavy metals with sorption capacity values of 562.1, 973.8, and 152.1 mg g^−1^ for Cd^2+^, Pb^2+^, and Cu^2+^, respectively, at 25 min with 10.0 mg dosage.^131^ Therefore, the prepared (SiO_2_@VB9) is considered as a promising material for the water remediation of such toxic heavy metals.

The modification of hollow magnetic silica nanoparticles (HMSMCs) with (3-mercaptopropyl)trimethoxysilane (MPTS) to form functionalized thiol nanoparticles (SH-HMSMCs) and its affinity to remove Hg^2+^ was studied. It was noted that the presence of the thiol group enhances the adsorption affinity toward Hg^2+^. Moreover, the adsorbent is easily recovered and reused.^132^ Also, the grafting of aminopropyltriethoxysilane on magnetic mesoporous SBA-15 microspheres and its capability for Pb^2+^ adsorption was investigated. The obtained data presented the efficient adsorption of Pb^2+^ over the prepared adsorbate with an adsorption capacity of 243.9 mg g^−1^.^133^

Amino-functionalized magnetic mesoporous silica (MSNs) was studied and examined as a sorbent to heavy metal ions (Pb^2+^, Cu^2+^, Cd^2+^) from its solutions. The adsorbate showed high adsorption performance with adsorption capacity reaching 289.7, 196.5, and 154.2 mg g^−1^ for Pb^2+^, Cu^2+^, and Cd^2+^, respectively.^134^ The surface of nanosilica prepared from rice husk with purity higher than 99% was modified with polydiallyl dimethylammonium chloride (PDADMAC) to form (PMNS) at pH 10 and 100 mM KCl. The results showed that PMNS is an effective adsorbent to remove beta-lactam cefixime (CEF) from actual hospital wastewater. The significant adsorption efficiency may be explained by electrostatic attractions between anionic CEF molecules and positively charged PMNS surfaces. Therefore, PMNS may be regarded as a novel sorbent to recover and separate antibiotics from wastewater.^135^*N*,*N*-Bis(salicylidene)-1,3-ethylenediamine Schiff base-functionalized mesoporous SBA-15 and decorated with Fe_3_O_4_ nanoparticles were fabricated to remove Ce(iii) ions. The composite shows high adsorption performance and excellent selectivity of Ce in the presence of various cations (La^3+^, Nb^3+^, Er^3+^, Cu^2+^, Cd^2+^, Cr^3+^, and Fe^2+^ ions).^136^

The decoration of surface silica nanoparticles with silver nanoparticles in the presence of protein as reducing and coating agents of the formed AgNPs was studied. The prepared composite shows high removal efficiency of the dye in both single and multicomponent systems. Moreover, it was found that the adsorption mainly occurs through electrostatic interaction, though π–π interactions, and pore diffusions. In addition, NSAgNPs illustrated long-term antibacterial activities *versus* both planktonic cells and biofilm of Gram-negative *Escherichia coli* and *Pseudomonas aeruginosa*. From the analysis of the data obtained from scanning electron and fluorescence microscopy, it is indicated that cell death takes place depending on irreversible damage of the cell membranes upon electrostatic interactions between positively charged sites of the composite and the negatively charged bacterial cell membranes.^137^ Amino and quaternary ammonium groups have functionalized the pores and the surface of MSNs, respectively, using post-synthesis and condensation techniques for drug delivery. The amino group-adsorbed Fe ions, which coordinated with bleomycin (BLM), was chosen as a model anti-cancer drug (NH_2_–Fe–BLM) in the pore surface. The quaternary ammonium groups in the surface of the particles were found to facilitate the penetration of nanoparticles into the cell.^138^ Also, the functionalization of MSNs by the amino group of (*N*-(2-aminoethyl)-3-aminopropyl methyl dimethoxysilane) was fabricated to remove Pb^2+^ ions from aqueous solutions. The experiments showed that the sorption reactions were feasible, spontaneous, and endothermic in nature. Furthermore, the nanomaterial can be regenerated by 0.01 M HNO_3_.^139^

Moreover, different functionalized silica nanomaterials have been used to capture and remove CO_2_ and other carbonyl compounds.^140–148^

Due to the differences in the metabolic reactions of antibiotic compounds in the human and animal bodies, considerable quantities of these compounds are not absorbed and leaves the bodies, and finally end in sewage systems and surface water, leading to harmful and toxicological effects on living organisms.^149–151^ Therefore, many works have been published to use silica-based nanomaterials to remove antibiotics from wastewater,^149–155^ where different parameters affecting the removal processes have been investigated. However, there is a noticeable lack of studies that treat the degradation and adsorption of antibiotics by SBA-15 nanomaterials type,^151^ highlighting the use of most nanomaterials in sorption processes.

It is important to note that in some cases, nanomaterials in environmental remediation applications and wastewater treatment face many challenges. It may form other contaminants themselves after being used, agglomeration, regeneration, and unstable under normal conditions. Thus, the employment of biodegradable nanomaterials is extremely interesting in environmental remediation applications and wastewater treatment applications.^117^ Increasing the utilization of suitable biodegradable nanomaterials in such fields will increase consumer confidence and contribute to the development of innovative technologies and be regarded as greener and secure alternative materials for these applications. Also, new technologies and the development of current technologies are required to overcome these challenges and improve the characteristics of nanomaterials by combining these new technologies with chemical and physical modification processes, taking into account the parameters required to produce novel nanomaterials such as cost-effectiveness, facile modification, greener, secure, non-poisoning, biodegradability, recyclability, and the possibility to recover.

## 3 Conclusion and future perspectives

This review shows that many interesting works and researches have been carried out to improve the properties of functionalized silica nanoparticles (SiO_2_ NPs) and develop their applications. SiO_2_ is the best choice of nanomaterial to use in various fields, and this is attributed to their superior features such as nanoscale size, high surface area, extensively porosity, high stability, excellent biocompatibility; the most valuable property of SiO_2_ is its ability to be merged with different materials (modifier) efficiently for potential applications. Five important applications (advanced catalysis, drug-delivery, biomedical applications, environmental remediation applications, and wastewater treatment) were selected for demonstrating the importance of the surface modification step in the various properties of the silica surface. The overview indicated that the functionalization of the silica surface has a positive effect, where the modification enhances the performance of the silica nanoparticles in different fields compared with unmodified ones. Moreover, this review provides simple and applicable modification strategies that are easy to set up without complicated experiments.

In addition, the combination of silica nanoparticles with magnetic nanoparticles will increase the performance of the modified silica part in different applications. Therefore, magnetic silica could be easily separated by the applied simple external magnetic field. Furthermore, these magnetic nanoparticles could be easily regenerated and reused, which will reduce the cost.

Recently, various studies have been done to study and apply nanomaterials and nanotechnology in advanced catalysis, drug delivery, biomedical applications, environmental remediation applications, and wastewater treatment, but many concerns are yet to be addressed. Therefore, further studies and more research are necessary to explain and develop the roles of functionalized silica nanoparticles (SiO_2_ NPs) in catalytic processes, cancer therapy, drug-delivery systems, environmental remediation processes, and wastewater treatment. There is hope for future perspectives that are required for the future utilization of these amazing nanocarriers in clinical applications as the first (SNPs) (Cornell dots) was approved for human clinical tests (currently, recruiting phase I and phase II trials).^104^ Although, the mechanisms by which the functionalized silica nanoparticles working in many fields are well known^117^ and have been discussed, its side-effects or degradation are underexplored, and the continuation of scientific studies is urgent to clarify the fate of these type of nanoparticles, especially in medical fields and environmental applications.

## Conflicts of interest

There are no conflicts to declare.

## Supplementary Material
